# Do disempowered bodies risk anaemia? Evidence from married women in Assam’s Sixth Schedule areas of Northeast India

**DOI:** 10.1080/16549716.2026.2612800

**Published:** 2026-01-12

**Authors:** Abigail Lalnuneng, Zothanchhingi Khiangte, Thiyam Seityajit Singh, Madhurima Samanta, Roshni Tripathy

**Affiliations:** a Department of Anthropology, Faculty of Science, University of Delhi, Delhi, India; b Department of English, Centre for Women Studies, Bodoland University, Kokrajhar, India

**Keywords:** women’s health, disempowerment, autonomy and empowerment, WAI, SWPER

## Abstract

**Background:**

Anaemia remains widespread among Indian women and reflects persistent structural and gendered inequities.

**Objective:**

This study examines how socio-demographic-economic, autonomy, and empowerment indicators interact to influence anaemia risk among married women in the Sixth Schedule districts of Assam, Northeast India.

**Methods:**

This study analysed 5245 married women aged 18–49 years from Assam’s seven Sixth Schedule districts using the NFHS–5 (2019–2021) data. Anaemia was modelled against sociodemographic-economic, autonomy, and empowerment indicators derived from contextually adapted indices (WAI-M and SWPER-M) using logistic regression to estimate unadjusted and adjusted odds ratios.

**Results:**

Anaemia affected 66.0% of women, ranging from 58.0% in Kokrajhar to 83.7% in Udalguri; women in Udalguri remained nearly five times more likely to be anaemic than those in Kokrajhar (AOR = 4.76; 95% CI = 2.46–9.24). Low decision-making power (AOR = 2.24; 95% CI = 1.28–3.94) and limited social independence (AOR = 3.09; 95% CI = 1.70–5.60) were significant predictors of anaemia. Counterintuitively, women with medium-high (AOR = 0.56; 95% CI = 0.32–1.00) and medium-low (AOR = 0.48; 95% CI = 0.26–0.86) tolerance of wife-beating were less likely to be anaemic than those categorised as highly empowered.

**Conclusions:**

Anaemia is not solely a biomedical condition but a manifestation of constrained autonomy, limited social independence, and unequal decision-making power. High-burden districts such as Udalguri and Baksa require structural interventions. Effective anaemia reduction necessitates a structural shift toward empowerment-centred interventions that integrate gender-transformative health and nutrition strategies.

## Paper Context

- **Main findings:** Anaemia among married women in Assam’s Sixth Schedule areas is rooted in structural disempowerment, manifested through limited autonomy, restricted decision-making, and constrained social independence.
- **Added knowledge:** Women’s agency mediates how structural hierarchies influence embodied health inequalities, with empowerment operating through thresholds and gradients, where limited autonomy increases anaemia risk but full assertion of agency may trigger social backlash in patriarchal contexts.
- **Global health impact for policy and action:** Reducing anaemia requires gender-transformative, empowerment-centred approaches addressing structural disempowerment, beyond behavioural or dietary interventions, supporting Sustainable Development Goals 2, 3, and 5.

## Background

Anaemia remains one of the world’s most persistent public health challenges, affecting over 1.9 billion people and contributing to 52 million years lived with disability in 2021 [1,2]. Defined as a reduction in haemoglobin concentration or red blood cell count impairing oxygen transport, anaemia disproportionately affects women in low- and middle-income countries (LMICs), particularly in South Asia, where prevalence among women is 35.7% [2,3]. In India, it is a deeply gendered condition. 53–57% of women aged 15–49 years are anaemic compared to 25% among men [4,5]. The burden is especially severe in the Northeast, where the prevalence of Anaemia among non-pregnant women rose from 38.7% in *National Family Health Survey-4 (*NFHS-4) to 50.1% in NFHS-5 [6]. In Assam, 66% of women are anaemic compared to 36% among men, with the highest prevalence among rural and socially marginalised women [5]. Within the Sixth Schedule districts – Kokrajhar, Dima Hasao, Karbi Anglong, and West Karbi Anglong, nearly 80% of women are affected, reflecting how anaemia persists as a symptom of structural inequality [7].

National programmes such as *Anaemia Mukt Bharat* and *POSHAN Abhiyaan* continue to frame anaemia largely as a biomedical or behavioural issue, emphasising supplementation, fortification, and dietary modification. While necessary, such approaches are insufficient in settings where gendered hierarchies constrain women’s capacity to act on health information or access care. Women’s health outcomes are shaped by their autonomy, their control over resources, mobility, and participation in decision-making and by their attitudes toward gender norms and violence [8–11]. Understanding anaemia, therefore, requires attention to the social structures and power relations that limit women’s agency.

The roots of these inequities lie in the long history of social stratification by gender, class, and ethnicity, which has institutionalised women’s disempowerment and limited their decision-making capacity [12,13]. Patriarchal norms have historically positioned women as subjects of male authority, thereby limiting their autonomy and reinforcing health disparities [9,14–16]. Feminist and decolonial scholars have expanded this perspective, demonstrating how women’s bodies, particularly those of Indigenous and Black women, have been controlled through intersecting systems of patriarchy, colonialism, and institutional domination [17–22]. Rather than a static attribute, empowerment is a relational and processual condition that emerges through negotiation, resistance, and reconstitution of power in daily life [15,23–25]. It is both outwardly enacted and internally realised, linking access to material, cognitive, and social resources with the capacity to exercise meaningful choice.

Empowerment is the crucible where conflicting and overlapping terms like choice, control, agency, and power intersect [26]. In Assam, these intersections are reflected in widespread gender-based violence: the state records India’s highest rate of crimes against women, 166 per 100,000 population, with 1678 rape cases in 2018 [27]. Power operates through material, social, and symbolic hierarchies reinforced by institutional arrangements that constrain both individual and collective agency [28,29]. Anaemia prevalence thus reflects not only proximate determinants such as diet, parity, and health knowledge but also structural inequalities in resource control and decision-making [10,11]. Contextual variables, including poverty, education, and access to services, further mediate these relationships [30–32].

Empowerment must therefore be examined as a multidimensional process encompassing intrapersonal, interpersonal, and structural domains [29,33]. Within the health context, it shapes women’s ability to act purposefully and participate in decision-making regarding healthcare, reproduction, and nutrition [34]. Women’s autonomy, a key dimension of empowerment, includes access to material and social resources, as well as control over bodily and domestic decisions [35–37]. However, even in communities often idealised as egalitarian, such as the tribal societies of Northeast India, autonomy remains uneven. Social isolation, low literacy, and economic marginalisation continue to restrict women’s choices [38]. These contradictions highlight that empowerment cannot be assumed from cultural stereotypes but must be interrogated within specific local contexts.

Government initiatives such as *Beti Bachao Beti Padhao*, *Pradhan Mantri Ujjwala Yojana*, *Mahila-e-Haat*, *Janani Suraksha Yojana*, and *Matru Vandana Yojana* have expanded access to education, employment, and healthcare [39,40]. Yet systemic inequalities persist, indicating that empowerment cannot be achieved through policy reform alone [41]. The persistence of anaemia among Indigenous and rural women reflects the failure to address gendered inequities embedded in structural and cultural systems.

Measuring empowerment in such settings requires tools sensitive to contextual realities. The Survey-based Women’s Empowerment (SWPER) Index [42,43] and the Women’s Autonomy Index [36,44] (WAI) provide robust frameworks for multidimensional autonomy and empowerment assessment but often overlook local nuances. To address this, the present study employs modified versions (WAI-M and SWPER-M) adapted to the sociocultural context of Northeast India.

The study focuses on married women because empowerment measures in NFHS-5 are available exclusively for this group. Moreover, marriage in the Indian sociocultural context constitutes a primary setting where gendered power, household decision-making, and access to resources are structured and negotiated. Concentrating on married women enables a conceptually coherent assessment of how household autonomy and spousal dynamics are associated with anaemia risk.

By operationalising autonomy and empowerment through these frameworks, this study examines the prevalence and determinants of anaemia among married women in Assam’s Sixth Schedule areas. Specifically, it investigates how variations in empowerment influence haemoglobin concentration and anaemia risk and whether empowerment mediates sociodemographic and economic disparities, thereby reframing anaemia as a manifestation of structural and gendered inequity rather than a purely biomedical disorder.

## Methods

### Study design and data source

This study utilised data from the Assam state sample of the NFHS-5, conducted between June 2019 and April 2021 as part of the Demographic and Health Survey (DHS) programme. The survey was implemented by the Ministry of Health and Family Welfare (MOHFW), Government of India (GoI), in collaboration with the International Institute for Population Sciences (IIPS), Mumbai. NFHS-5 employed a two-stage stratified sampling design, with villages (rural) and Census Enumeration Blocks (urban) as primary sampling units (PSUs), followed by systematic random sampling of households within each PSU. The study’s objective was explanatory inference rather than population-level prevalence estimation; therefore survey weights were not applied in primary regression analyses. Following NFHS/DHS analytical practice, key design covariates, such as district, rural/urban residence, and household socioeconomic status, were included to account for differential sampling across strata.

#### Sample selection and inclusion criteria

**Figure 1.**
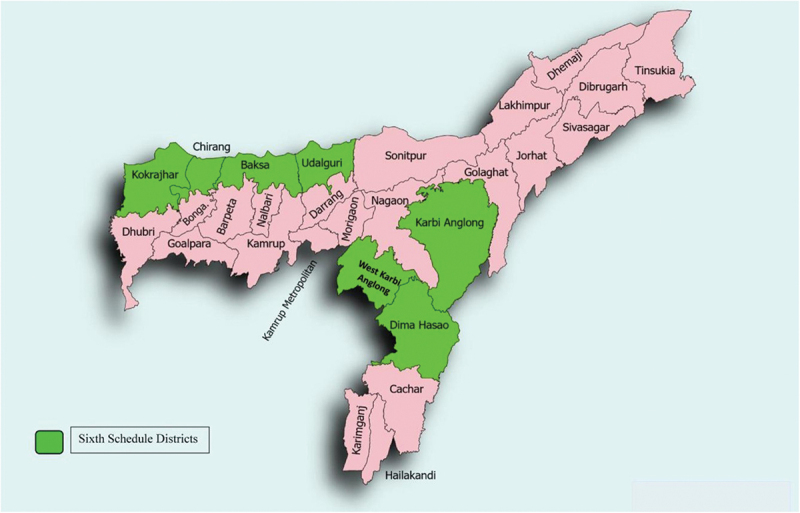
Map of Sixth Schedule areas of Assam.

The study focused on Assam’s Sixth Schedule districts, including Kokrajhar, Dima Hasao, Chirang, Baksa, Udalguri, Karbi Anglong, and West Karbi Anglong, administered under special constitutional provisions for tribal populations (Figure 1). Of 7499 women aged 15–49 years, those < 18 years (*n* = 726), unmarried (*n* = 1,090), pregnant (*n* = 259), or with missing haemoglobin or key explanatory variables (*n* = 179) were excluded. Analyses used complete-case observations for each model, the effective sample size therefore varies slightly across models (Tables 1 and 2). Missing data were minimal and unlikely to bias estimates.

**Table 1.** General characteristics (socio-demographic and economic characteristics) in relationto haemoglobin concentration and anaemia among married women in Sixth Schedule areas of Assam.

Background Characteristics	n (%)	Haemoglobin Concentration (g/L) Mean ± SD	Anaemia No n (%)	Anaemia Yes n (%)
**District**				
Kokrajhar	765 (14.6)	114.91 ± 14.17	321 (42.0)	444 (58.0)
Dima Hasao	654 (12.5)	116.16 ± 13.35	265 (40.5)	389 (59.5)
Chirang	791 (15.1)	112.71 ± 14.81	277 (35.0)	514 (65.0)
Baksa	861 (16.4)	110.18 ± 14.26	226 (26.2)	635 (73.8)
Udalguri	762 (14.5)	106.13 ± 13.23	124 (16.3)	638 (83.7)
Karbi Anglong	690 (13.2)	114.69 ± 13.50	271 (39.3)	419 (60.7)
West Karbi Anglong	722 (13.8)	115.16 ± 12.43	286 (39.6)	436 (60.4)
***F and χ^2^ value, p value***		F = 50.46, p < 0.001*	χ^2^ = 18.33, p < 0.001*	
**Type of Place of Residence**				
Urban	427 (8.1)	114.60 ± 14.26	167 (39.1)	260 (60.9)
Rural	4818 (91.9)	112.52 ± 14.09	1603 (33.3)	3215 (66.7)
***t and χ^2^ value, p value***		t = 2.92, p = 0.004*	χ^2^ = 5.98, p = 0.014*	
**Social Category**				
Scheduled Caste	680 (15.4)	112.16 ± 14.62	226 (33.2)	454 (66.8)
Scheduled Tribe	2393 (54.3)	113.08 ± 13.82	825 (34.6)	1568 (65.5)
OBC	1008 (22.9)	110.98 ± 14.32	293 (29.1)	715 (70.9)
None of them	330 (7.5)	112.82 ± 14.15	111 (33.6)	219 (66.4)
***F and χ^2^ value, p value***		F = 5.46, p = 0.001*	χ^2^ = 9.49, p = 0.023*	
**Native Language**				
Assamese	2777 (52.9)	111.75 ± 14.17	858 (30.9)	1919(69.1)
Bengali	549 (10.5)	114.62 ± 14.66	214 (39.0)	335(61.0)
Hindi	125 (2.4)	115.70 ± 12.60	53 (42.4)	72(57.6)
Other	1794 (32.3)	113.35 ± 13.84	645 (35.1)	1149(64.0)
***F and χ^2^ value, p value***		F = 10.82, p < 0.001*	χ^2^ = 24.91, p < 0.001*	
**Religion**				
Hindu	3905 (74.5)	112.14 ± 14.02	1256 (32.2)	2649 (67.8)
Muslim	718 (13.7)	114.81 ± 14.18	287 (40.0)	431 (60.0)
Christian	581 (11.1)	113.52 ± 14.31	210 (36.1)	371 (63.9)
Other	41 (0.7)	115.44 ± 15.66	17 (41.5)	24 (58.5)
***F and χ^2^ value, p value***		F = 8.58, p < 0.001*	χ^2^ = 19.41, p < 0.001*	
**Family Size**				
< 5	2579 (49.2)	112.59 ± 14.32	864 (33.5)	1715 (66.5)
≥5	2666 (50.8)	112.78 ± 13.92	906 (34.0)	1760 (66.0)
***t and χ^2^ value, p value***		*t = −0.48, p = 0.6*34	*χ^2^ = 0.14, p = 0.712*	
**Cohabitation Duration (years)**				
0–4	781 (14.9)	111.39 ± 13.56	227 (29.1)	554 (70.9)
5–9	1088 (20.7)	112.75 ± 13.69	365 (33.6)	723 (66.4)
10–14	1009 (19.2)	112.99 ± 14.19	351 (34.8)	658 (65.2)
15–19	897 (17.1)	113.61 ± 14.24	332 (37.0)	565 (63.0)
20–24	760 (14.5)	113.42 ± 14.36	275 (36.2)	485 (63.8)
25–29	503 (9.6)	111.98 ± 14.80	165 (32.8)	338 (67.2)
≥30	207 (3.9)	110.76 ± 14.53	55 (26.6)	152 (73.4)
***F and χ^2^ value, p value***		F = 3.02, p = 0.006*	χ^2^ = 19.43, p = 0.003*	
**Age Group (years)**				
18–29	1783 (34.0)	112.02 ± 13.81	560 (31.4)	1223 (68.6)
30–39	1908 (36.4)	113.43 ± 14.19	687 (36.0)	1221 (64.0)
40–49	1554 (29.6)	112.55 ± 14.34	523 (33.7)	1031 (66.3)
***F and χ^2^ value, p value***		F = 4.71, p = 0.009*	χ^2^ = 8.72, p = 0.013*	
**Husband’s Age**				
18–29	102 (13.1)	113.64 ± 11.97	36 (35.3)	66 (64.7)
30–39	287 (36.9)	112.41 ± 13.74	92 (32.1)	195 (67.9)
40–49	249 (32.0)	113.63 ± 14.24	95 (38.2)	154 (61.8)
50–59	123 (15.8)	113.11 ± 14.57	42 (34.2)	81 (65.8)
60–68	17 (2.2)	115.29 ± 11.96	6 (35.3)	11 (64.7)
***F and χ^2^ value, p value***		*F = 0.42, p = 0.796*	*χ^2^ = 2.22, p = 0.695*	
**Wealth Index**				
Poorest	1027 (19.6)	111.69 ± 14.14	319 (31.1)	708 (68.9)
Poorer	1124 (21.4)	112.22 ± 14.09	372 (33.1)	752 (66.9)
Middle	1161 (22.1)	113.48 ± 14.12	416 (35.8)	745 (64.2)
Richer	1057 (20.2)	112.40 ± 14.11	340 (32.2)	717 (67.8)
Richest	876 (16.7)	113.75 ± 14.05	323 (36.9)	553 (63.1)
***F and χ^2^ value, p value***		F = 3.86, p = 0.004*	χ^2^ = 10.79, p = 0.029*	
**Highest Education (Respondent)**				
No education	1375 (26.2)	111.75 ± 14.45	441 (32.1)	934 (67.9)
Primary	855 (16.3)	112.45 ± 14.40	281 (32.9)	574 (67.1)
Secondary	3015 (57.5)	113.18 ± 13.86	1048 (34.8)	1967 (65.2)
***F and χ^2^ value, p value***		F = 5.00 p = 0.007*	*χ^2^ = 3.40, p = 0.182*	
**Respondent Currently Working**				
No	664 (81.4)	112.78 ± 13.73	221 (33.3)	443 (66.7)
Yes	152 (18.6)	114.55 ± 13.75	60 (39.5)	92 (60.5)
***F and χ^2^ value, p value***		*t = −1.44, p = 0.150*	*χ^2^ = 2.10, p = 0.147*	
**Respondent Occupation**				
Not Working	648 (79.4)	112.71 ± 13.66	213 (32.9)	435 (67.1)
Agricultural	62 (7.6)	113.90 ± 11.76	22 (35.5)	40 (62)
Skilled/unskilled manual	46 (5.6)	116.07 ± 14.63	21 (45.7)	25 (54.3)
Other	60 (7.4)	114.32 ± 15.66	25 (41.7)	35 (58.3)
***F and χ^2^ value, p value***		*F = 1.12, p = 0.341*	*χ^2^ = 4.89, p = 0.196*	
**Respondent Works For**				
For family member	129 (76.8)	114.36 ± 13.99	54 (41.9)	75 (58.1)
For someone else	17 (10.1)	113.47 ± 11.30	5 (29.4)	12 (70.6)
Self-employed	22 (13.1)	117.18 ± 16.05	9 (40.9)	13 (59.1)
***F and χ^2^ value, p value***		*F = 0.44, p = 0.642*	*χ^2^ = 0.97, p = 0.616*	
**Type of Earnings from Respondent’s Work**				
Not paid	24 (14.3)	114.92 ± 10.62	5 (20.8)	19 (79.2)
Cash only	123 (73.2)	114.52 ± 14.90	54 (43.9)	69 (56.1)
Cash and in-kind	21 (12.5)	115.05 ± 12.22	9 (42.9)	12 (57.1)
***F and χ^2^ value, p value***		*F = 0.02, p = 0.982*	*χ^2^ = 4.49, p = 0.106*	
**Husband’s Occupation**				
Agricultural	335 (41.1)	115.10 ± 12.92	130 (38.8)	205 (61.2)
Skilled/unskilled manual	216 (26.5)	115.45 ± 12.78	95 (44.0)	121 (56.0)
Other	265 (32.5)	114.89 ± 12.78	98 (37.0)	167 (63.0)
***F and χ^2^ value, p value***		*F = 0.11, p = 0.893*	*χ^2^ = 2.58, p = 0.275*	
**Husband/Partner’s Education**				
No education	176 (21.5)	112.11 ± 12.82	54 (30.7)	122 (69.3)
Primary	133 (16.3)	112.20 ± 15.72	47 (35.3)	86 (64.7)
Secondary & Higher	507 (62.2)	113.69 ± 13.74	180 (35.5)	327 (64.5)
***F and χ^2^ value, p value***		*F = 1.21, p = 0.299*	*χ^2^ = 1.40, p = 0.496*	

*represents a statistical significance of p < 0.05.

**Table 2.** Women autonomy and empowerment in relation to haemoglobin concentration and anaemia among married women in Assam’s Sixth Schedule areas.

Women Autonomy and Empowerment	n (%)	Haemoglobin Concentration (g/L) Mean ± SD	Anaemia No n (%)	Anaemia Yes n (%)
**Women’s Autonomy According to WAI**				
High autonomy (7–10)	595 (72.9)	113.41 ± 13.80	210 (35.3)	385 (64.7)
Moderate autonomy (4–6)	204 (25.0)	112.75 ± 13.63	69 (33.8)	135 (66.2)
Low autonomy (0–3)	17 (2.1)	106.76 ± 12.08	2 (11.8)	15 (88.2)
***F and χ^2^ value, p value***	*F = 2.03, p = 0.132*		*χ^2^ = 4.10, p = 0.129*	
**Women’s Autonomy According to WAI-M**				
High Autonomy ( > 8.0)	164 (20.1)	112.52 ± 14.799	58 (35.4)	106 (64.6)
Moderate High Autonomy (7.1–8.0)	173 (21.2)	115.22 ± 13.617	76 (43.9)	97 (56.1)
Moderate Low Autonomy (5.5–7.0)	255 (31.3)	113.02 ± 13.018	80 (31.4)	175 (68.6)
Low Autonomy ( < 5.5)	224 (27.5)	112.01 ± 13.749	67 (29.9)	157 (70.1)
***F and χ^2^ value, p value***		*F = 1.95, p = 0.120*	χ^2^ = 10.06, p = 0.018*	
**Women’s Empowerment According to SWPER Previous Study**				
**Decision-Making Empowerment**				
High empowerment	711 (13.6)	113.51 ± 13.77	257 (36.2)	454 (63.8)
Medium empowerment	293 (5.6)	112.61 ± 14.63	92 (31.4)	201 (68.6)
Low empowerment	4241 (80.9)	112.56 ± 14.14	1421 (33.5)	2820 (66.5)
***F and χ^2^ value, p value***		*F = 1.38, p = 0.251*	*χ^2^ = 2.66, p = 0.264*	
**Attitude toward Violence Empowerment**				
High empowerment	5135 (97.9)	112.67 ± 14.11	1730 (33.7)	3405 (66.3)
Medium empowerment	110 (2.1)	113.50 ± 14.59	40 (36.4)	70 (63.6)
***t and χ^2^ value, p value***		*t = −0.61, p = 0.542*	*χ^2^ = 0.34, p = 0.557*	
**Social Independence Empowerment**				
High empowerment	1781 (34.0)	112.90 ± 13.94	612 (34.4)	1169 (65.6)
Medium empowerment	1935 (36.9)	112.42 ± 14.14	640 (33.1)	1295 (66.9)
Low empowerment	1529 (29.2)	112.78 ± 14.31	518 (33.9)	1011 (66.1)
***F and χ^2^ value, p value***		*F = 0.56, p = 0.57*	*χ^2^ = 0.70, p = 0.703*	
**Women Empowerment According to SWPER-M**				
**Decision making**				
High Empowerment	135 (17.6)	114.76 ± 13.448	55 (40.7)	80 (59.3)
Medium-high Empowerment	240 (31.2)	114.7 ± 13.08	94 (39.2)	146 (60.8)
Medium-low Empowerment	202 (26.3)	111.8 2 ± 15.041	67 (33.2)	135 (66.8)
Low Empowerment	192 (25.0)	111.34 ± 13.27	52 (27.1)	140 (72.9)
***F and χ^2^ value, p value***		F = 3.38, p = 0.018*	χ^2^ = 9.38, p = 0.025*	
**Attitude towards violence**				
High Empowerment	133 (17.3)	111.33 ± 13.5	33 (24.8)	100 (75.2)
Medium-high Empowerment	239 (31.1)	113.9 ± 13.624	88 (36.8)	151 (63.2)
Medium-low Empowerment	191 (24.8)	113.66 ± 14.194	77 (40.3)	114 (59.7)
Low Empowerment	206 (26.8)	112.85 ± 13.793	70 (34)	136 (66)
***F and χ^2^ value, p value***		***F = 1.12, p = 0.339***	χ^2^ = 8.89, p = 0.031*	
**Social Independence**				
High Empowerment	84 (10.9)	117.88 ± 12.036	43 (51.2)	41 (48.8)
Medium-high Empowerment	275 (35.8)	111.55 ± 14.824	92 (33.5)	183 (66.5)
Medium-low Empowerment	130 (16.9)	113.14 ± 14.172	47 (36.2)	83 (63.8)
Low Empowerment	280 (36.4)	113.21 ± 12.76	86 (30.7)	194 (69.3)
***F and χ^2^ value, p value***		F = 4.59, p = 0.003*	χ^2^ = 12.32, p = 0.006*	

### Data collection

*Haemoglobin concentration* was measured using *HemoCue* analysers by trained field personnel. *Anaemia* was defined as haemoglobin < 120 g/L (altitude and smoking adjusted) and classified as mild (100–119 g/L), moderate (80–109 g/L), or severe ( < 80 g/L) following World Health Organization, 2024 (WHO) guidelines [45].

*Sociodemographic and Socioeconomic Variables* included district, rural/urban residence, social category (Scheduled Tribe (ST), Scheduled Caste (SC), Other Backward class (OBC), or none, religion, native language, age group (18–29, 30–39, 40–49 years), and family size ( < 5 or ≥ 5 members). Education and occupation were coded for both respondents and husbands. The household wealth index derived via principal component analysis (PCA) of assets was categorised into quintiles (poorest to richest). Employment status, type of work, and type of earnings were included.

### Measurement of women autonomy and empowerment

Modified Women’s Autonomy Index (WAI-M) expanded the conventional index [44,46,47] to capture graded and relational aspects of autonomy across three domains:

1. *Decision-Making Autonomy (4 items)*: Includes personal healthcare, large household purchases, visits to relatives, and use of husband’s earnings. Responses were ordinally scored (0 = husband alone, 0.5 = joint, 1 = respondent alone).
2. *Asset and Financial Autonomy (4 items)*: Ownership of house and land coded as 0 = none, 0.5 = joint, 1 = sole; ownership of bank account and mobile phone coded binarily (0/1).
3. *Mobility Autonomy (4 items)*: Ability to travel to markets, health facilities, or outside the village, and perceived difficulty in seeking medical help, similarly scored (0–1).

The final 12-item scale demonstrated acceptable internal consistency (Cronbach’s α = 0.64). Scores (0–12) were divided into quartiles representing low to high autonomy.

#### Modified survey-based women’s empowerment index (SWPER-M)

Grounded in Kabeer’s (1999) [15] conceptualisation of empowerment as the capacity to make *strategic life choices that affect their health outcomes*, the SWPER-M retained three core domains [42,43] but was adapted for the present study, including 16 items in total.

1. *Decision-making autonomy (5 items):* This domain measured the respondent’s active participation in household and financial decisions, reflecting agency in personal, familial, and economic spheres. In addition to the previous three items, two more items are added which includes decision on spending respondent’s own earnings and decision on spending husband’s earnings. The coding for the modified and previous study remains the same: 1 = Husband/other alone, 0 = Joint decision, 1 = Respondent alone
2. *Attitude toward violence (5 items):* The women’s attitude towards domestic violence and their perceptions of sexual autonomy remain the same in the modified SWPER. The coding for the modified and previous study remains the same: 1 = Yes (violence justified); 0 = Don’t know; 1 = No (violence not justified)
3. *Social independence (6 items):* This domain captured dimensions of social exposure, access to information, education, and life-course experiences contributing to women’s independence and awareness. A total of six items were included, with two new items usually allowed to go to the health facility, and usually allowed to go to places outside the respondent’s village, coded as: 1 = Not at all; 0 = Someone else; 1 = Alone. The coding for frequency of reading newspapers or magazines remains the same for the previous and modified SWPER. Continuous variables such as respondents’ highest education attained (in years), age at first birth, and age at first cohabitation were included in their original numeric form to capture gradient variation in social independence.

#### Scoring and validation

The SWPER-M index demonstrated acceptable internal consistency (Cronbach’s α = 0.70). Sampling adequacy was confirmed by the Kaiser – Meyer – Olkin measure (0.750), and Bartlett’s test of sphericity was significant (χ^2^ = 786.62, *p* < 0.001), supporting suitability for PCA. PCA was used to extract components and group items into three domains: decision-making, attitude toward violence, and social independence. Domain scores were calculated using weighted item loadings, normalised across individuals, and standardised to allow comparability. Standardised scores were then classified into four levels of empowerment based on interquartile ranges (IQRs).

### Statistical analysis

All data analyses were performed using IBM SPSS Statistics for Windows, Version 28.0. A p-value < 0.05 was considered statistically significant for all inferential tests. Descriptive statistics summarised the socio-demographic, economic, and empowerment-related characteristics of participants. Continuous variables were presented as mean ± SD, and categorical variables as frequencies and percentages.

The dependent variable was anaemia status (anaemic/non-anaemic). Independent variables included socio-demographic and economic factors, along with indicators of women’s autonomy and empowerment, derived from both the original WAI and SWPER indices, and their modified versions developed for the present study. Bivariate analyses examined the distribution of haemoglobin concentration and anaemia prevalence across explanatory variables. Independent sample *t*-tests and one-way analysis of variance (ANOVA) were used to test mean differences in haemoglobin concentration between groups, while Pearson’s chi-square (χ^2^) tests assessed associations between categorical variables and anaemia status.

To identify independent determinants of anaemia, binary logistic regression analyses were conducted in two stages. *Model 1* included socio-demographic and economic predictors to estimate their adjusted effects on anaemia. *Model 2* incorporated autonomy and empowerment indicators (from both original and modified indices), controlling for significant covariates from *Model 1*, to assess the independent contribution of women’s autonomy and empowerment to anaemia risk. Both unadjusted odds ratios (ORs) and adjusted odds ratios (AORs) with 95% confidence intervals (CIs) were reported. Model fit was evaluated using the Hosmer – Lemeshow goodness-of-fit test, with all models demonstrating acceptable fit (*p* > 0.05).

## Results

### Socio-demographic-economic profile and anaemia prevalence

**Figure 2.**
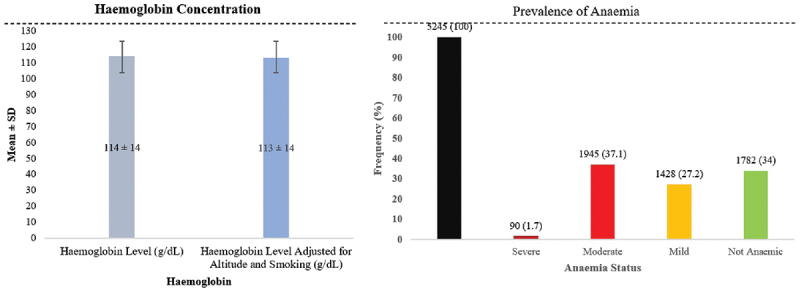
Anaemia levels and haemoglobin concentrations among women in Assam’s Sixth Schedule districts.

Anaemia was highly prevalent among married women in Assam’s Sixth Schedule areas, with an overall prevalence of 66.3%. The mean haemoglobin concentration was 113 ± 14 g/L (altitude- and smoking-adjusted). Moderate anaemia accounted for 36.8% of cases, mild anaemia for 27.2%, and severe anaemia for 1.8% (Figure 2). Anaemia prevalence and haemoglobin concentration varied significantly across several socio-demographic and economic factors (Table 1).

Significant inter-district variation was observed in mean haemoglobin concentration (F = 50.46, *p* < 0.001) and anaemia prevalence (χ^2^ = 183.33, *p* < 0.001). Women in Udalguri had the lowest mean haemoglobin (106.13 ± 13.23 g/L) and the highest anaemia prevalence (83.7%), followed by Baksa (73.8%), Chirang (65%), Karbi Anglong and West Karbi Anglong (61%), whereas women in Dima Hasao (116.16 ± 13.35 g/L, 59.5%) and Kokrajhar (114.91 ± 14.17 g/L, 58.0%) showed higher mean haemoglobin concentration and lower prevalence of anaemia. Rural residence was associated with lower mean haemoglobin (112.52 ± 14.09 g/L) and higher anaemia prevalence (66.7%) compared with urban areas (114.60 ± 14.26 g/L; 60.9%) (*t* = 2.92, *p* < 0.01; χ^2^ = 5.98, *p* < 0.05).

Mean haemoglobin differed significantly by social category (F = 5.46, *p < 0.01*), lowest among OBCs (110.98 ± 14.32 g/L) with the highest anaemia prevalence (70.9%), followed by SCs (112.16 ± 14.62 g/L, 68.5%) and STs (113.08 ± 13.82 g/L, 65.5%) women (χ^2^ = 9.49, *p < 0.05*). Language differences were significant (F = 10.82, *p < 0.001*; χ^2^ = 24.91, *p < 0.001*), with native Assamese-speaking women showing lower mean haemoglobin (111.75 ± 14.17 g/L) and higher anaemia prevalence (69.1%), while native Hindi speakers had higher mean haemoglobin (115.70 ± 12.60 g/L) and lower anaemia prevalence (57.6%). Religious differences followed a similar pattern (F = 8.58, *p < 0.001*; χ^2^ = 19.41, *p < 0.001*), with Hindu women (112.23 ± 14.23 g/L; 67.8%) showing higher anaemia prevalence than Muslim (114.84 ± 13.09 g/L; 60.0%) and Christian (114.03 ± 13.46 g/L; 63.9%) participants.

Mean haemoglobin decreased with longer marital cohabitation (F = 3.02, *p < 0.01*). Women cohabiting for ≥30 years had the lowest mean value (110.76 ± 14.53 g/L) and highest anaemia prevalence (73.4%), compared with those living with their husbands for < 10 years (112.96 ± 13.77 g/L; 65.2%) and 10–19 years (113.33 ± 14.14 g/L; 65.2%) (χ^2^ = 19.43, *p < 0.01*). Younger women (18–29) years show lower mean haemoglobin (111.92 ± 14.05 g/L) and higher anaemia prevalence (68.6%) than those aged 30–39 years (113.63 ± 13.81 g/L; 64.0%) and 40–49 years (113.37 ± 14.84 g/L; 66.1%) (F = 4.71, *p < 0.01*; χ^2^ = 8.72, *p < 0.05*).

Haemoglobin and anaemia varied significantly by household wealth index (F = 3.86, *p < 0.01*; χ^2^ = 10.79, *p < 0.05*). Women in the poorest quintile had lowest mean haemoglobin (111.26 ± 14.48 g/L) and higher anaemia prevalence (68.9%) compared with the richest group (114.71 ± 13.73 g/L; 63.1%). Education level showed a significant association with haemoglobin concentration (F = 5.00, *p < 0.01*) but not with anaemia prevalence (χ^2^ = 3.40, *p* > 0.05).

No significant differences were found in haemoglobin or anaemia status by family size, currently working, occupation, type of earnings, and husband’s occupation or education (all *p > 0.05*).

### Women’s autonomy, empowerment and anaemia prevalence

Associations between women’s autonomy and empowerment indices (WAI, SWPER, and modified versions) and haemoglobin/anaemia outcomes are shown in Table 2.

#### Women’s autonomy (WAI and WAI-M)

Based on WAI (previous study), mean haemoglobin differences and anaemia prevalence across autonomy levels were non-significant (F = 2.03, *p > 0.05*; χ^2^ = 4.098, *p > 0.05*), but trends suggested that women with low autonomy had poorer outcomes (106.76 ± 12.08 g/L; 88.2%) than those with moderate (112.75 ± 13.63 g/L; 66.2%) or high autonomy (113.41 ± 13.80 g/L; 64.7%). With the WAI-M, a more nuanced pattern emerged. Anaemia prevalence differed significantly by autonomy (χ^2^ = 10.06, *p < 0.05*), although differences in mean haemoglobin were not significant (F = 1.95, *p > 0.05*). Women with low autonomy ( < 5.5) had lower haemoglobin (112.01 ± 13.75 g/L) and higher anaemia (70.1%) than those with moderate high autonomy (7.1–8.0; 115.22 ± 13.62 g/L; 56.1%), indicating that the modified index was more sensitive to empowerment gradients.

#### Women’s empowerment (SWPER and SWPER-M)

The SWPER index (previous study) showed no statistically significant association with anaemia. The SWPER-M, however, revealed robust associations. In the decision-making domain, women with low empowerment had lower haemoglobin (111.34 ± 13.27 g/L) and higher anaemia (72.9%) than highly empowered women (114.76 ± 13.45 g/L; 59.3%) (F = 3.38, *p < 0.05*; χ^2^ = 9.38, *p < 0.05*). Under social independence, haemoglobin and anaemia varied significantly (F = 4.59, *p < 0.01*; χ^2^ = 12.32, *p < 0.01*): the *lowest empowerment group* had the *highest anaemia prevalence* (69.3%) and lower haemoglobin (113.21 ± 12.76 g/L), while highly empowered women had higher haemoglobin (117.88 ± 12.04 g/L) and lower anaemia (48.8%). Although the attitude-to-violence domain did not affect haemoglobin (F = 1.12, *p > 0.05*), its association with anaemia was significant (χ^2^ = 8.89, *p > 0.05*). Counterintuitively, women with high empowerment (rejecting violence) had lower haemoglobin and higher anaemia prevalence (111.33 ± 13.50 g/L; 75.2%), while those with medium-high empowerment showed higher haemoglobin and lower anaemia prevalence (113.90 ± 13.62 g/L; 63.2%).

Across indices, low autonomy and empowerment were consistently associated with lower haemoglobin concentration and higher anaemia prevalence, with modified indices (WAI-M and SWPER-M) appearing more sensitive than their previous counterparts, and capturing the subtle relationship between women’s empowerment and anaemia outcomes more effectively.

#### Item-wise analysis of women’s autonomy and empowerment dimensions

Item-level analysis (Supplementary Table S1) provides deeper insight into the specific dimensions of autonomy and empowerment linked to low haemoglobin and anaemia. Within the previous WAI framework, women excluded from healthcare decision-making had lower haemoglobin (111.23 ± 13.54 g/L) and higher anaemia prevalence (74.7%) than those with high decision-making authority (113.40 ± 13.79 g/L; χ^2^ = 4.37, *p* < 0.05). Similarly, lack of control over visits to family or relatives and restrictions on mobility (such as visiting health facilities or travelling outside the village) were significantly associated with lower haemoglobin and higher anaemia (*p* < 0.05 for all). Similar trends persisted for WAI-M, women whose husbands solely decided on household expenditure had significantly poorer outcomes (110.90 ± 13.50 g/L; 73.2%) than those who participated jointly (F = 3.47, *p* < 0.05; χ^2^ = 6.12, *p* < 0.05). Restricted access to health facilities was also significantly associated with lower haemoglobin (F = 2.04, *p* < 0.05). Under the SWPER-M, the social independence domain was particularly notable, women who read newspapers at least once a week had higher haemoglobin levels (115.28 ± 14.30 g/L) and significantly lower anaemia prevalence (56.6%; χ^2^ = 6.286, *p* < 0.05).

Both the composite and item-level analyses demonstrate a consistent pattern: Low autonomy and empowerment are significantly associated with lower haemoglobin concentrations and higher anaemia prevalence.

### Determinants of anaemia

Binary logistic regression (Table 3) identified key determinants of anaemia. After adjusting for autonomy and empowerment, all the socio-demographic and economic associations lost significance except for districts, suggesting mediation through empowerment.

**Table 3.** Unadjusted and adjusted logistic regression estimates for socio-demographic and economic factors associated with anaemia among married women in Assam’s Sixth Schedule areas.

Socio-demographic and economic characteristics	OR (95% C.I.)	*p* value	AOR (95% C.I.)	*p* value
**District**				
*Kokrajhar (Reference)*	–	–		
Dima Hasao	1.06 (0.86 − 1.31)	*0.583*	1.35 (0.77–2.38)	*0.292*
**Chirang**	**1.34 (1.09 − 1.65)**	0.005*	1.37 (0.79–2.39)	*0.265*
**Baksa**	**2.03 (1.65 − 2.50)**	< 0.001*	**2.34 (1.31–4.17)**	0.004*
**Udalguri**	**3.72 (2.93 − 4.73)**	< 0.001*	**4.76 (2.46–9.24)**	< 0.001*
Karbi Anglong	1.12 (0.91 − 1.38)	*0.298*	1.20 (0.68–2.11)	*0.533*
West Karbi Anglong	1.10 (0.90 − 1.36)	*0.357*	1.13 (0.64–1.97)	*0.679*
**Type of place of residence**				
*Urban (Reference)*	–	–		
**Rural**	**1.29 (1.05 − 1.58)**	0.015*	1.26 (0.76 − 2.08)	*0.368*
**Social Category**				
None of them (Reference)	–	–		
Schedule caste	1.02 (0.77 − 1.34)	*0.899*	1.00 (0.46 − 2.17)	*0.998*
Schedule tribe	0.96 (0.76 − 1.23)	*0.764*	0.93 (0.47 − 1.83)	*0.830*
OBC	1.24 (0.95 − 1.61)	*0.117*	0.83 (0.40 − 1.72)	*0.620*
**Native language**				
*Hindi (Reference)*	–	–		
**Assamese**	**1.65 (1.14 − 2.37)**	0.007*	1.40 (0.51–3.83)	*0.517*
Bengali	1.15 (0.78 − 1.71)	*0.481*	1.42 (0.49–4.11)	*0.518*
Other	1.31 (0.91 − 1.89)	*0.148*	1.61 (0.58–4.44)	*0.360*
**Religion**				
*Other (Reference)*	–	–		
Hindu	1.49 (0.80 − 2.79)	*0.208*	1.25 (0.44–3.59)	*0.679*
Muslim	1.06 (0.56 − 2.02)	*0.85*	0.79 (0.26–2.43)	*0.680*
Christian	1.25 (0.66 − 2.38)	*0.495*	0.97 (0.30–3.12)	*0.965*
**Cohabitation duration (Years)**				
0–4 (Reference)	–	–		
5–9	**0.81 (0.66 − 0.99)**	0.04*	0.84 (0.50–1.43)	*0.528*
10–14	**0.77 (0.63 − 0.94)**	0.01*	0.90 (0.52–1.56)	*0.700*
15–19	**0.70 (0.57 − 0.86)**	0.001*	0.59 (0.34–1.02)	*0.058*
20–24	**0.72 (0.58 − 0.90)**	0.003*	0.99 (0.54–1.82)	*0.981*
25–29	0.84 (0.66 − 1.07)	*0.156*	0.76 (0.40–1.47)	*0.419*
30+	1.13 (0.80 − 1.60)	*0.48*	1.02 (0.40–2.64)	*0.961*
**Age Group (years)**				
30–39 *Reference)*	–	–		
18–29	**1.32 (1.13 − 1.53)**	< 0.001*	0.92 (0.64–1.33)	*0.657*
40–49	1.08 (0.93 − 1.25)	*0.341*	0.94 (0.64–1.37)	*0.751*
**Wealth Index**				
*Richest (Reference)*	–	–		
Poorest	**1.30 (1.07 − 1.57)**	0.008*	0.96 (0.58–1.60)	*0.875*
Poorer	1.18 (0.98 − 1.42)	*0.079*	0.99 (0.60–1.62)	*0.955*
Middle	1.05 (0.87 − 1.26)	*0.629*	0.89 (0.55–1.45)	*0.645*
Richer	**1.23 (1.02 − 1.49)**	0.03*	0.98 (0.60–1.62)	*0.948*

*Note:* AORs are adjusted for modified Women’s Autonomy Index (WAI) and modified SWPER domains (Decision-Making, Attitude Toward Violence, and Social Independence). Variables were selected based on chi-square analysis (p < 0.05). OR = Odds Ratio, AOR = Adjusted Odds Ratio, C.I. = Confidence Interval, * = Statistically significant.

A significant district gradient was evident, with Udalguri (83.7%) emerging as the most affected area. Women in Udalguri were nearly four times more likely to be anaemic than those in Kokrajhar (OR = 3.72, 95% CI [2.93–4.73], *p* < 0.001), and this association strengthened after adjustment (AOR = 4.76, 95% CI [2.46–9.24], *p* < 0.001). Baksa also showed a significantly elevated risk (AOR = 2.34, 95% CI [1.31–4.17], *p* = 0.004), while in Chirang, the initially higher risk (OR = 1.34, 95% CI [1.09–1.65], *p* = 0.005) became non-significant after adjustment (AOR = 1.37, 95% CI [0.79–2.39], *p* = 0.265). No significant differences were found for Dima Hasao, Karbi Anglong, or West Karbi Anglong in either model.

For other variables (residence, language, marital duration, age, wealth, social category, and religion), the associations with anaemia observed in the unadjusted models lost statistical significance after adjustment for autonomy and empowerment. No significant associations were found with social category or religion with anaemia in either model, suggesting no measurable difference by social category or religion in the present sample.

Table 4 further underscores the pivotal role of women’s empowerment dimensions in shaping anaemia risk. The WAI-M showed no significant association with anaemia in either the unadjusted or adjusted models, while the SWPER-M *decision-making power* and *social independence* domains exhibited significant and most consistent associations with anaemia. Women with limited participation in household decisions and constrained social autonomy faced significantly higher odds of anaemia, even after adjusting for socio-demographic and economic factors. In the adjusted model, those with l*ow decision-making empowerment* had more than double the risk of anaemia compared with *highly empowered women* (AOR = 2.24; 95% CI: 1.28–3.94; *p < 0.01*). *Social independence* showed a strong graded relationship, wherein medium-high (AOR = 2.38; 95% CI: 1.34–4.24; *p < 0.01*), medium-low (AOR = 2.35; 95% CI: 1.21–4.57; *p < 0.05*), and low empowerment levels (AOR = 3.09; 95% CI: 1.70–5.60; *p < 0.001*) were progressively associated with higher odds of anaemia, a pattern that remained significant after adjustment, underscoring restricted participation in household decision-making and limited social independence as independent risk factors beyond sociodemographic and economic disadvantage.

**Table 4.** Unadjusted and adjusted logistic regression estimates for women’s autonomy and empowerment indicators associated with anaemia among married women in Assam’s Sixth Schedule areas.

*Women’s Autonomy and Empowerment Factors*	OR (95% C.I.)	*p value*	AOR (95% C.I.)	*p value*
**WAI-M**				
**Modified WAI (IQR)**				
High Autonomy (Reference)	–	–	–	–
Moderate-high Autonomy	0.70 (0.45 − 1.08)	*0.109*	0.90 (0.55–1.50)	*0.693*
Moderate-low Autonomy	1.20 (0.79 − 1.81)	*0.396*	1.21 (0.75–1.96)	*0.435*
Low Autonomy	1.28 (0.84 − 1.97)	*0.256*	1.42 (0.85–2.38)	*0.180*
**SWPER-M**				
**Decision Making**				
High empowerment (Reference)	–	–		
Medium high empowerment	1.07 (0.70–1.64)	*0.765*	1.31 (0.79–2.18)	*0.300*
Medium low empowerment	1.38 (0.88–2.18)	*0.157*	1.52 (0.88–2.60)	*0.132*
Low empowerment	**1.85 (1.16–2.96)**	0.010*	**2.24 (1.28–3.94)**	0.005*
**Attitude to Violence**				
High empowerment (Reference)	–	–		
Medium high empowerment	**0.57 (0.35–0.91)**	0.018*	**0.56 (0.32–1.00)**	0.050*
Medium low empowerment	**0.49 (0.30–0.80)**	0.004*	**0.48 (0.26–0.86)**	0.014*
Low empowerment	0.64 (0.39–1.04)	*0.074*	0.69 (0.38–1.25)	*0.218*
**Social Independence**				
High empowerment (Reference)				
Medium high empowerment	**2.09 (1.27–3.42)**	0.004*	**2.38 (1.34–4.24)**	0.003*
Medium low empowerment	**1.85 (1.06–3.25)**	0.030*	**2.35 (1.21–4.57)**	0.012*
Low empowerment	**2.37 (1.44–3.89)**	0.001*	**3.09 (1.70–5.60)**	0.000*

***Note**:* AORs are adjusted for sociodemographic and economic characteristics, including District, Type of Place of Residence, Social Category, Native Language, Religion, Cohabitation Duration (years), Age Group (years), and Wealth Index. Variables were selected based on chi-square analysis (p < 0.05). OR = Odds Ratio, AOR = Adjusted Odds Ratio, C.I. = Confidence Interval, * = Statistically significant.

Interestingly, for *attitude towards violence*, women with *high empowerment* (those strongly rejecting all forms of wife-beating), those with l*ower empowerment* exhibited lower odds of anaemia. In both unadjusted and adjusted models, women with medium-high (AOR = 0.56; 95% CI: 0.32–1.00; *p = 0.050)* and medium-low empowerment (AOR = 0.48; 95% CI: 0.26–0.86; *p < 0.05*) were significantly less likely to be anaemic compared with the *high-empowerment* reference group. Although the association for the *low-empowerment* group was not statistically significant (AOR = 0.69; 95% CI: 0.38–1.25; *p > 0.05*), the direction of effect remained protective.

## Discussion

This study documents markedly low mean haemoglobin (113 ± 14 g/L) and a critical anaemia prevalence, affecting two-thirds (66%) of married women across Assam’s Sixth Schedule areas. These levels exceed both state and national NFHS-5 estimates [48], indicating that decades of nutrition and health programmes have been unsuccessful in reaching the structural roots of the problem.

### District-level inequalities in anaemia

Udalguri stands out as a critical hotspot, with an alarming anaemia prevalence of 83.7%, the highest among all districts and a 27% increase since NFHS-4. Women in Udalguri are nearly five times more likely to be anaemic than those in Kokrajhar (AOR = 4.76, 95% CI: 2.46–9.24, *p* < 0.001), and risk persists even after adjustment for socioeconomic and empowerment factors, highlighting structural and ecological vulnerabilities, rather than individual choices. Udalguri’s concentration of tea estate labourers, dependent on phytate-rich diets, excessive tea consumption, and haemoglobinopathies such as *HbE* and *β-thalassaemia*, could intensify their nutritional and genetic susceptibility [49,50]. Baksa shows a similar, though slightly less extreme pattern, women remain twice as likely to be anaemic as in Kokrajhar, reflecting parallel disadvantages. In contrast, the initially high anaemia risk in Chirang disappeared after adjustment for autonomy and empowerment, suggesting gendered autonomy and empowerment as mediating mechanisms. Similar association has been documented across India, Bangladesh, and Ethiopia, where women’s empowerment, reflected in decision-making power and attitudes toward violence, significantly reduces anaemia risk [11,43,51].

Although Dima Hasao showed no significant difference in the adjusted model, the steep rise from 39.4% to 61.1% highlights emerging vulnerability and potential transitional phase towards high anaemia burden. Karbi Anglong and West Karbi Anglong districts maintain a relatively stable but high prevalence (~60%), suggesting entrenched barriers related to geographic isolation, poor healthcare access, and infrastructural deprivation [52,53]. Even Kokrajhar, with the lowest prevalence (58%), exceeds the WHO threshold for moderate public health concern [45,54], confirming that anaemia remains a systemic and structural challenge across all districts.

### Mediation of sociodemographic and economic effects through autonomy and empowerment

Bivariate results initially suggested that anaemia was more prevalent among rural residents, Assamese-speaking women, the younger and recently married, and those in poorer households, patterns widely observed across South Asia and other LMICs [55–62]. However, these associations disappeared after introducing autonomy and empowerment indicators, revealing that sociodemographic and economic disparities operate primarily through the mediating pathways of women’s autonomy, agency, and social independence rather than as independent determinants.

The attenuation of place, wealth, and age effects underscores a unifying mechanism, in which structural constraints on women’s decision-making and social independence are associated with spatial and economic inequalities influencing embodied adverse health outcomes. Rural disadvantage, for instance, reflects not geographical isolation alone but the gendered organisation of labour, restricted mobility, and dependence within patriarchal household systems [63–67]. Similarly, wealth gradients disappear once empowerment is introduced, indicating that material assets improve women’s health when coupled with agency over income and expenditure [68,69]. Age and marital duration follow comparable trajectories: younger and recently married women, often positioned lowest within kinship hierarchies, face limited autonomy, constrained healthcare access, and heavy reproductive workloads, all of which increase anaemia risk [70,71]. Linguistic identity intersects with these hierarchies. The initially higher anaemia odds among native Assamese-speaking women dissipated after adjustment, suggesting that language is associated with deeply embedded patriarchal relations rather than an independent determinant of health.

Across these contexts, kinship-based authority structures, gendered food hierarchies, and social dependence collectively mediate how sociodemographic position shapes women’s health status [72–74]. Empirically, the disappearance of statistical significance once autonomy and empowerment indices were introduced reflects a structural mediation effect: women’s agency transforms how age, wealth, or residence are associated with anaemia vulnerability. In other words, it is not *where* women live or *how much* they own, but how much power they exercise over their bodies, movement, and household decisions that determines anaemia outcomes. This pattern reinforces feminist and anthropological insights that material and demographic variables acquire health relevance only through gendered social structures [15,23,75].

### Women autonomy and empowerment: thresholds, gradients, and counterintuitive patterns in the determinants of anaemia

Autonomy and empowerment emerged as decisive yet contextually mediated determinants of anaemia. Although the WAI-M indicated that women with low autonomy were 1.42 times more likely to be anaemic, this association attenuated after adjusting for socioeconomic and demographic variables, a pattern also observed in Nepal and Ethiopia, where autonomy effects were stronger at collective rather than individual levels [71,76]. This highlights that autonomy is relational and structured by household hierarchies and gendered norms rather than an individual attribute [12,15].

Empowerment, as captured through the SWPER-M Index, revealed domain-specific yet structurally interconnected associations with anaemia risk. Women with *limited decision-making autonomy* and *low social independence* faced significantly higher odds of anaemia than those with higher autonomy. The association displayed two distinct patterns: a *threshold effect* for decision-making and a *graded, dose – response relationship* for social independence. Specifically, women with the *lowest decision-making power* faced nearly double the risk of anaemia, while those with *lower social independence* showed a progressive increase in risk, reaching nearly threefold higher among the least independent.

These patterns underscore that the health benefits of empowerment accrue most significantly when women cross a minimal threshold of autonomy and social freedom. Below this threshold, restricted decision-making severely limits access to food, healthcare, and household resources, while constrained social independence reflects entrenched structural barriers shaped by education, mobility, and exposure to information, influencing health literacy, autonomy in service utilisation, and negotiation power within social institutions. The persistence of these associations, even after adjusting for socioeconomic and demographic variables, underscores that empowerment exerts an independent influence on women’s health beyond material deprivation.

Comparable results from sub-Saharan Africa and India reaffirm that low social independence consistently predicts higher anaemia [77–79]. Cross-contextual findings from Tanzania and multi-country reviews [80,81] underscore that the transformative effects of empowerment are most visible when women move from *disempowerment to basic agency*, gaining minimal decision-making rights, education, and mobility rather than in marginal improvements among already empowered groups. Consistent with recent findings in India, social independence exerts protective effects across caste and tribal groups, marking it as a robust and cross-cutting determinant of anaemia [78].

These results reveal that empowerment is not a homogeneous or static construct but a stratified and context-dependent process negotiated through everyday power relations. *Decision-making autonomy* shapes immediate, household-level control over resources, whereas *social independence* reflects broader structural freedoms that sustain long-term health gains. This pattern aligns with feminist conceptions of empowerment as a relational and structural process [15,24], wherein agency is not confined to individual autonomy but materialises through the transformation of social and institutional structures that perpetuate women’s subordination. It also resonates with Wallerstein’s [9] (1992) and Rappaport’s [28] (1987) argument that empowerment must be understood not merely as individual agency but as collective resistance to structural domination.

Women’s *attitudes toward domestic violence* (a proxy for internalised gender norms) and anaemia risk revealed a counterintuitive pattern. Compared with women in the *highest empowerment group* (who rejected all justifications of wife-beating), those with *medium-high empowerment* had about 44% lower odds of anaemia (AOR ≈0.56), and those with *medium-low empowerment* had roughly 52% lower odds (AOR ≈0.48). In contrast, women with *low empowerment* (who largely justify wife-beating) showed about 36% lower odds but did not differ significantly in anaemia risk from the highly empowered group. Although this pattern may appear to indicate a protective effect of moderate empowerment, it is more plausibly interpreted as a reflection of *structural and relational constraints* rather than a genuine advantage of conforming to restrictive gender norms.

*Medium-low-and-high empowered* women may strategically navigate patriarchal expectations by balancing partial conformity with limited assertion of agency. Such negotiation enables them to maintain spousal cooperation and social acceptance, thereby sustaining access to material and emotional resources, including food, healthcare, and household support, factors known to buffer against anaemia [10,11,31]. Yet this cannot be equated with genuine empowerment, it reveals how women sustain wellbeing within structures that still constrain their agency. This should not be misread as the benefit of subordination but as evidence of *adaptive compliance*: women’s strategic negotiation of social acceptance to secure material stability, food, and healthcare within restrictive gender orders. As feminist theorists argue, power operates not only through coercion but through internalised adaptation to inequality, reproducing gender hierarchies that shape women’s health outcomes [17,28,29].

The apparent protective trend among the *least empowered* likely reflects *dependency-based stability* maintained through male financial provisions or control over household resources, rather than health advantage. Once the confounding influence of socioeconomic position is accounted for, the illusion of protection collapses, revealing anaemia not as a reflection of individual compliance, but as an outcome of structural subordination that restricts women’s decision-making power, mobility, and health autonomy [9,12,13,82].

Conversely, women with *high empowerment* (those who unequivocally reject all forms of wife-beating) appear to face increased anaemia risk. This seemingly counterintuitive finding can be attributed to the *social backlash effect* of empowerment in rigidly patriarchal contexts. Assertive women may encounter spousal resistance, social ostracism, or restricted access to household resources, all of which elevate psychosocial stress and limit their nutritional and health autonomy [14–16,22]. Women’s bodies, particularly those of Indigenous and marginalised communities, often become sites where intersecting systems of patriarchy, colonialism, and class domination exert control and discipline [17,18,20,21,83] that regulate their agency and health.

The observed patterns thus challenge instrumentalist views of empowerment as a linear pathway to improved health. Empowerment, as both a condition and a struggle, is relational, context-specific, and contested. *Medium-low-high empowerment* may offer short-term protection by accommodating patriarchy, while *full empowerment* incurs social penalties until structural hierarchies are transformed. Anaemia in Assam’s Sixth Schedule areas, therefore, represents not merely nutritional deprivation but the embodied consequence of constrained autonomy and empowerment within stratified systems of power, a call for interventions that expand collective, not only individual, agency. The counterintuitive nature of empowerment thus reflects not women’s failure to act, but the punitive structures that constrain their right to do so, which in turn affects their health outcomes.

## Conclusion

Anaemia among married women in Assam’s Sixth Schedule areas reflects deep-seated structural inequities in autonomy, decision-making, and social independence, rather than isolated individual behaviours. The disappearance of socioeconomic and demographic effects after accounting for autonomy and empowerment indicates that anaemia is embedded within gendered systems of power. Empowerment operates through thresholds and gradients: limited decision-making power and social independence are associated with heightened vulnerability to anaemia, while higher levels of asserted agency may trigger social backlash within patriarchal contexts, revealing a counterintuitive nature of empowerment where women’s pursuit of autonomy can itself incur health penalties until structural hierarchies are transformed.

Udalguri and Baksa districts, where anaemia is most severe, require structural interventions that move beyond household level behavioural change, addressing labour conditions, workplace nutrition, screening for haemoglobinopathies, and local food systems, alongside sustained empowerment strategies.

An *empowerment-centred interpretation* of anaemia, aligned with Sustainable Development Goals (SDG) 2, 3, and 5, is critical for addressing anaemia. Programmes and interventions narrowly focused on nutrition, poverty alleviation, or behavioural change will remain inadequate without addressing the relational dimensions of power that shape women’s health and well-being. The study reveals not isolated vulnerabilities but a coherent system of *structural subordination* in which empowerment constitutes both the pathway and the solution to anaemia reduction. Advancing health equity in Assam’s Sixth Schedule areas requires reframing empowerment not as women’s adaptation to patriarchy, but as its transformation.

### Strengths and limitations

This study’s strengths include population-based sampling across all Sixth Schedule districts of Assam and use of a multidimensional empowerment index. Limitations include cross-sectional design limiting causal inference, and reliance on self-reported autonomy and empowerment measures that may not fully capture collective, or less visible forms of power and may be susceptible to social desirability bias, particularly in socially constrained and marginalised contexts. Although general dietary information was available, the absence of detailed micronutrient data (including iron, folate and vitamin B12) and the lack of biomarkers of inflammation limited our ability to distinguish between iron deficiency anaemia and anaemia of chronic disease or other underlying conditions, including haemoglobinopathies.

## Supplementary Material

Supplementary Tables clean version.docx

STROBE_checklist_Do Disempowered Bodies Risk Anaemia.docx

## Data Availability

The data is publicly available in an open-access repository, and the corresponding author can also share it upon reasonable request. The data used in this study can be accessed from The DHS Program repository at- https://dhsprogram.com/data/available-datasets.cfm.
