# Correction

**DOI:** 10.1080/16549716.2026.2683305

**Published:** 2026-06-09

**Article title:** Do disempowered bodies risk anaemia? Evidence from married women in Assam’s Sixth Schedule areas of Northeast India

**Authors:** Abigail Lalnuneng, Zothanchhingi Khiangte, Thiyam Seityajit Singh, Madhurima Samanta and Roshni Tripathy

**Journal:** *Global Health Action*

**Bibliometrics:** Volume 19, Number 1, 2612800

**DOI:** https://doi.org/10.1080/16549716.2026.2612800

When this article was originally published, the adjusted odds ratio (AOR) value for the “medium-high” category was missing.

The sentence appeared as: “Counterintuitively, women with medium-high (AOR=95% CI=0.56; 0.32–1.00) and medium-low (AOR=0.48; 95% CI=0.26–0.86) tolerance of wife-beating were less likely to be anaemic than those categorised as highly empowered.”

This has now been corrected to: “Counterintuitively, women with medium-high (AOR=0.56; 95% CI=0.32–1.00) and medium-low (AOR=0.48; 95% CI=0.26–0.86) tolerance of wife-beating were less likely to be anaemic than those categorised as highly empowered.”

