# Supplementary material for: Do disempowered bodies risk anaemia? Evidence from married women in Assam’s Sixth Schedule areas of Northeast India
Source: Glob Health Action. 2026 Jan 12;19(1):2612800. doi: 10.1080/16549716.2026.2612800 (PMC12798656; doi:10.1080/16549716.2026.2612800)
Supplement: Supplementary Tables clean version.docx [file ZGHA_A_2612800_SM0030.docx]

**Supplementary Table 1:** Item-wise Analysis of Women’s Autonomy and Empowerment Indices (WAI and SWPER) in Relation to Haemoglobin Concentration and Anaemia among Married Women in the Sixth Schedule Areas of Assam.

| **Women Autonomy and Empowerment** | **n (%)** | **Haemoglobin Concentration (g/L) Mean ± SD** | **Anaemia**  **No**  **n (%)** | **Anaemia**  **Yes**  **n (%)** |
| --- | --- | --- | --- | --- |
| **WAI Previous Study (10 items)** | | | | |
| **Decision Making Power Dimension (3 Items)** | | | | |
| ***1.*** ***Person who usually decides on respondent’s health care*** | | | | |
| Respondent not involved | 95 (12.2) | 111.23 ± 13.54 | 24 (25.3) | 71 (74.7) |
| Respondent involved | 683 (87.8) | 113.40 ± 13.79 | 247 (36.2) | 436 (63.8) |
| *t and χ² value, p value* | *t = -1.44, p = 0.151* | | ***χ² = 4.37, p = 0.037**** | |
| ***2.*** ***Person who usually decides on large household purchases*** | | | | |
| Respondent not involved | 118 (15.2) | 111.37 ± 13.38 | 33 (28.0) | 85 (72.0) |
| Respondent involved | 660 (84.8) | 113.45 ± 13.82 | 238 (36.1) | 422 (63.9) |
| *t and χ² value, p value* | *t = -1.51, p = 0.132* | | *χ² = 2.89, p = 0.089* | |
| ***3.*** ***Person who usually decides on visits to family or relatives*** | | | | |
| Respondent not involved | 84 (10.8) | 110.14 ± 12.5 | 17 (20.2) | 67 (79.8) |
| Respondent involved | 694 (89.2) | 113.50 ± 13.88 | 254 (36.6) | 440 (63.4) |
| *t and χ² value, p value* | ***t = -2.11, p = 0.035**** | | ***χ² = 8.84, p = 0.003***** | |
| **Assets and Ownership Dimension (4 Items)** | | | | |
| ***4.*** ***Owns House*** | | | | |
| Owns house | 358 (43.9) | 112.92 ± 13.93 | 120 (33.5) | 238 (66.5) |
| Does not own | 458 (56.1) | 113.35 ± 13.60 | 161 (35.2) | 297 (64.8) |
| *t and χ² value, p value* | *t = -0.34, p = 0.734* | | *χ² = 0.24, p = 0.626* | |
| ***5.*** ***Owns Bank Account*** | | | | |
| No bank account | 144 (17.6) | 114.37 ± 13.70 | 58 (40.3) | 86 (59.7) |
| Has bank account | 672 (82.4) | 112.84 ± 13.74 | 223 (33.2) | 449 (66.8) |
| *t and χ² value, p value* | *t = 1.21, p = 0.225* | | *χ² = 2.64, p = 0.104* | |
| ***6.*** ***Owns Land*** | | | | |
| Does not own land | 519 (63.6) | 113.10 ± 13.90 | 181 (34.9) | 338 (65.1) |
| Owns land | 297 (36.4) | 113.06 ± 13.48 | 100 (33.7) | 197 (66.3) |
| *t and χ² value, p value* | *t = 0.04, p = 0.97* | | *χ² = 2.08, p = 0.354* | |
| ***7.*** ***Owns Mobile Phone*** | | | | |
| No mobile phone | 351 (43.0) | 112.51 ± 13.62 | 110 (31.3) | 241 (68.7) |
| Owns mobile phone | 465 (57.0) | 113.56 ± 13.83 | 171 (36.8) | 294 (63.2) |
| *t and χ² value, p value* | *t = -1.08, p = 0.279* | | *χ² = 2.62, p = 0.106* | |
| **Mobility Dimensions (3 Items)** | | | | |
| ***8.*** ***Usually allowed to go to market*** | | | | |
| Respondent not involved | 69 (8.5) | 111.39 ± 12.29 | 19 (27.5) | 50 (72.5) |
| Respondent involved | 747 (91.5) | 113.27 ± 13.86 | 262 (35.1) | 485 (64.9) |
| *t and χ² value, p value* | *t = -1.08, p = 0.279* | | *χ² = 1.59, p = 0.207* | |
| ***9.*** ***Usually allowed to go to health facility*** | | | | |
| Respondent not involved | 52 (6.4) | 109.5 ± 12.08 | 11 (21.2) | 41 (78.8) |
| Respondent involved | 764 (93.6) | 113.35 ± 13.82 | 270 (35.3) | 494 (64.7) |
| *t and χ² value, p value* | *t = -1.96, p = 0.05* | | ***χ² = 4.34, p = 0.037**** | |
| ***10.*** ***Usually allowed to go outside the village*** | | | | |
| Respondent not involved | 37 (4.5) | 109.62 ± 12.49 | 7 (18.9) | 30 (81.1) |
| Respondent involved | 779 (95.5) | 113.27 ± 13.78 | 274 (35.2) | 505 (64.8) |
| *t and χ² value, p value* | *t = -1.58, p = 0.114* | | ***χ² = 4.13, p = 0.042***** | |
| **WAI-M (12 items)** | | | | |
| **Decision Making Power (4 Items)** | | | | |
| ***1.*** ***Person who usually decides on respondent’s health care*** | | | | |
| Respondent alone | 71 (9.1) | 113.17 ± 14.62 | 24 (33.8) | 47 (66.2) |
| Respondent and husband/partner | 612 (78.7) | 113.42 ± 13.70 | 223 (36.4) | 389 (63.6) |
| Husband/partner alone | 95 (12.2) | 111.23 ± 13.54 | 24 (25.3) | 71 (74.7) |
| *F and χ² value, p value* | *F = 1.04, p = 0.353* | | *χ² = 4.56, p = 0.102* | |
| ***2.*** ***Person who usually decides on large household purchases*** | | | | |
| Respondent alone | 24 (3.1) | 114.21 ± 12.88 | 11 (45.8) | 13 (54.2) |
| Respondent and husband/partner | 636 (81.7) | 113.42 ± 13.87 | 227 (35.7) | 409 (64.3) |
| Husband/partner alone | 118 (15.2) | 111.37 ± 13.38 | 33 (28.0) | 85 (72.0) |
| *F and χ² value, p value* | *F = 1.18, p = 0.309* | | *χ² = 3.94, p = 0.140* | |
| ***3.*** ***Person who usually decides on visits to family or relatives*** | | | | |
| Respondent alone | 51 (6.6) | 114.84 ± 12.85 | 24 (47.1) | 27 (52.9) |
| Respondent and husband/partner | 643 (82.6) | 113.39 ± 13.96 | 230 (35.8) | 413 (64.2) |
| Husband/partner alone | 84 (10.8) | 110.14 ± 12.50 | 17 (20.2) | 67 (79.8) |
| ***F and χ² value, p value*** | ***F = 2.49, p = 0.083*** | | ***χ² = 11.49, p = 0.003**** | |
| **Addition Decision Making Power** | | | | |
| ***4.*** ***Person who usually decides what to do with money husband earns*** | | | | |
| Respondent alone | 29 (3.8) | 117.24 ± 13.03 | 13 (44.8) | 16 (55.2) |
| Respondent and husband/partner | 593 (76.9) | 113.50 ± 13.85 | 216 (36.4) | 377 (63.6) |
| Husband/partner alone | 149 (19.3) | 110.90 ± 13.50 | 40 (26.8) | 109 (73.2) |
| *F and χ² value, p value* | ***F = 3.47, p = 0.032**** | | ***χ² = 6.12, p = 0.047**** | |
| **Asset and Ownership (4 Items)** | | | | |
| **5.** ***Owns Bank Account (same as WAI Previous)*** | | | | |
| **6.** ***Owns Mobile Phone (same as WAI Previous)*** | | | | |
| ***7.*** ***Owns House*** |  |  |  |  |
| Does not own | 458 (56.1) | 113.25 ± 13.6 | 161 (35.2) | 297 (64.8) |
| Owns alone | 218 (26.7) | 113.39 ± 13.46 | 77 (35.3) | 141 (64.7) |
| Owns jointly | 140 (17.2) | 112.20 ± 14.66 | 43 (30.7) | 97 (69.3) |
| *F and χ² value, p value* | *F = 0.37, p = 0.690* | | *χ² = 1.04, p = 0.595* | |
| ***8.*** ***Owns land*** | | | | |
| Does not own | 519 (63.6) | 113.10 ± 13.9 | 181 (34.9) | 338 (65.1) |
| Owns alone | 170 (20.8) | 113.33 ± 12.89 | 60 (35.3) | 110 (64.7) |
| Owns jointly | 127 (15.6) | 112.95 ± 13.94 | 40 (31.5) | 87 (68.5) |
| *F and χ² value, p value* | *F = 0.03, p = 0.972* | | *χ² = 0.59, p = 0.746* | |
| **Mobility (4 Items)** | | | | |
| ***9.*** ***Usually allowed to go to the market*** | | | | |
| Not at all | 69 (8.5) | 111.39 ± 12.29 | 19 (27.5) | 50 (72.5) |
| Alone | 500 (61.3) | 113.67 ± 14.14 | 187 (37.4) | 313 (62.6) |
| With someone else only | 247 (30.3) | 112.44 ± 13.27 | 75 (30.4) | 172 (69.6) |
| *F and χ² value, p value* | *F = 1.25, p = 0.287* | | *χ² = 5.21, p = 0.074* | |
| ***10.*** ***Usually allowed to go to the health facility*** | | | | |
| Not at all | 52 (6.4) | 109.50 ± 12.08 | 11 (21.2) | 41 (78.8) |
| Alone | 379 (46.4) | 113.10 ± 14.30 | 140 (37.0) | 239 (63.0) |
| With someone else only | 385 (47.2) | 113.60 ± 13.34 | 130 (33.8) | 255 (66.2) |
| *F and χ² value, p value* | *F = 2.04, p = 0.013** | | *χ² = 5.19, p = 0.075* | |
| ***11.*** ***Usually allowed to go to places outside this village*** | | | | |
| Not at all | 37 (4.5) | 100.62 ± 12.49 | 7 (18.9) | 30 (81.1) |
| Alone | 422 (51.7) | 113.45 ± 13.54 | 154 (36.5) | 268 (63.5) |
| With someone else only | 357 (43.8) | 113.12 ± 13.99 | 120 (33.6) | 237 (66.4) |
| *F and χ² value, p value* | *F = 1.30, p = 0.273* | | *χ² = 4.84, p = 0.089* | |
| ***12.*** ***Permission to go - Getting medical help (initially included in structural barriers)*** | | | | |
| No problem | 3772 (71.9) | 112.38 ± 14.13 | 1244 (33.0) | 2528(67) |
| Big problem | 571 (10.9) | 113.37 ± 13.93 | 204 (35.7) | 367(64.3) |
| Not a big problem | 902 (71.2) | 113.55 ± 14.13 | 322 (35.7) | 580(64.3) |
| *F and χ² value, p value* | ***F = 3.27, p = 0.038**** | | *χ² = 3.53, p = 0.171* | |
| **SWPER- M (16 items)** | | | | |
| **Decision making power (5 Items)** | | | | |
| ***1.*** ***Person who usually decides on respondent’s health care (Same as WAI-M)*** | | | | |
| ***2.*** ***Person who usually decides on large household purchases Same as WAI-M)*** | | | | |
| ***3.*** ***Person who usually decides on visits to family or relatives Same as WAI-M)*** | | | | |
| ***4.*** ***Person who usually decides what to do with money husband earns Same as WAI-M)*** | | | | |
| ***5.*** ***Person who usually decides how to spend respondent’s earnings*** | | | | |
| Respondent alone | 16 (10.4) | 119.63 ± 15.34 | 10 (62.5) | 6 (37.5) |
| Respondent and husband/partner | 96 (76.8) | 114.72 ± 14.31 | 43 (44.8) | 53 (55.2%) |
| Husband/partner alone | 13 (12.8) | 107.15 ± 16.81 | 4 (30.8) | 9 (69.2) |
| *F and χ² value, p value* | *F = 2.60, p =0.078* | | *χ² = 3.02, p = 0.221* | |
| **Attitude toward Violence (5 items)** | | | | |
| ***6.*** ***Beating justified if wife goes out without telling husband*** | | | | |
| Yes | 55 (6.7) | 110.55 ± 15.36 | 17 (30.9) | 38 (69.1) |
| No | 761 (93.3) | 113.29 ± 13.61 | 264 (34.7) | 497 (65.3) |
| *t and χ² value, p value* | *t = -1.43, p = 0.152* | | *χ² = 0.32, p = 0.569* | |
| ***7.*** ***Beating justified if wife neglects children*** | | | | |
| Yes | 71 (8.7) | 112.82 ± 12.80 | 24 (33.8) | 47 (66.2) |
| No | 744 (91.2) | 113.13 ± 13.84 | 257 (34.5) | 487 (65.5) |
| *t and χ² value, p value* | *t = -0.18, p = 0.853* | | *χ² = 0.54, p = 0.763* | |
| ***8.*** ***Beating justified if wife argues with husband*** | | | | |
| Yes | 79 (9.7) | 112.46 ± 12.42 | 23 (29.1) | 56 (70.9) |
| No | 735 (90.3) | 113.15 ± 13.88 | 257 (35.0) | 478 (65.0) |
| *t and χ² value, p value* | *t = -0.43, p = 0.668* | | *χ² = 1.08, p = 0.298* | |
| ***9.*** ***Beating justified if wife refuses sex*** | | | | |
| Yes | 34 (4.2) | 111.44 ± 15.05 | 12 (35.3) | 22 (64.7) |
| No | 773 (94.7) | 113.18 ± 13.73 | 267 (34.5) | 506 (65.5) |
| *t and χ² value, p value* | *t = -0.72, p = 0.471* | | *χ² = 0.61, p = 0.737* | |
| ***10.*** ***Beating justified if wife burns food*** | | | | |
| Yes | 39 (4.8) | 113.10 ± 11.50 | 10 (25.6) | 29 (74.4) |
| No | 772 (95.2) | 113.09 ± 13.86 | 271 (35.1) | 501 (64.9) |
| *t and χ² value, p value* | *t = 0.01, p = 0.994* | | *χ² = 1.40, p = 0.236* | |
| **Social Independence (3 items + 3 continuous)** | | | | |
| ***11.*** ***Usually allowed to go to the health facility (same as WAI-Modified Mobility Dimension)*** | | | | |
| ***12.*** ***Usually allowed to go to places outside this village (same as WAI-Modified Mobility Dimension)*** | | | | |
| ***13.*** ***Frequency of reading newspaper/magazine*** | | | | |
| Not at all | 4553 (86.8) | 112.62 ± 14.09 | 1523 (33.5) | 3030 (66.5) |
| Less than once a week | 547 (10.4) | 112.60 ± 14.28 | 184 (33.6) | 363 (66.4) |
| At least once a week | 145 (2.8) | 115.28 ± 14.30 | 63 (43.4) | 82 (56.6) |
| *F and χ² value, p value* | *F = 2.52, p = 0.081* | | ***χ² = 6.29, p = 0.043***** | |
| **Continuous Variables** | | | ***Pearson Correlation, p value*** | |
| ***Education in single years*** | 6.01 ± 4.43 | | -0.015, p > 0.05 | |
| ***Age at first birth*** | 21.06 ± 4.09 | | 0.002, p > 0.05 | |
| ***Age at first cohabitation*** | 19.58 ± 4.27 | | -0.010, p > 0.05 | |

** = Statistically significant at p<0.05*
